# Shotgun-metagenomics reveals a highly diverse and communal microbial network present in the drains of three beef-processing plants

**DOI:** 10.3389/fcimb.2023.1240138

**Published:** 2023-09-08

**Authors:** Vignesh Palanisamy, Joseph M. Bosilevac, Darryll A. Barkhouse, Sarah E. Velez, Sapna Chitlapilly Dass

**Affiliations:** ^1^ Department of Animal Science, Texas A&M University, College Station, TX, United States; ^2^ U. S. Department of Agriculture, Roman L. Hruska U.S. Meat Animal Research Center, Clay Center, Lincoln, NE, United States; ^3^ Molecular Center of Excellence, Invisible Sentinel, bioMerieux Inc., Philadelphia, PA, United States

**Keywords:** beef-processing plants, biofilms, shotgun metagenomics, core microbiome, antimicrobial resistance, horizontal gene transfer

## Abstract

**Background:**

Multi-species biofilms pose a problem in various environments, especially food-processing environments. The diversity of microorganisms in these biofilms plays a critical role in their integrity and protection against external biotic and abiotic factors. Compared to single-species biofilms, mixed-species biofilms are more resistant to various stresses, including antimicrobials like sanitizers. Therefore, understanding the microbiome composition and diversity in biofilms and their metabolic potential is a priority when developing intervention techniques to combat foodborne pathogens in food processing environments.

**Methods:**

This study aimed to describe and compare the microbiome profile of 75 drain biofilm samples obtained from five different locations (Hotscale, Hotbox, Cooler, Processing, & Grind room) of three beef-processing plants (Plant A, B & C) taken over two timepoints 2017-18 (T1) and 2021 (T2) by shotgun sequencing

**Results:**

Core microbiome analysis found Pseudomonas, Psychrobacter, and Acinetobacter to be the top three prevalent genera among the plants and locations. Alpha diversity analysis demonstrated a high diversity of microbiome present in all the plants and locations across the time points. Functional analysis showed the high metabolic potential of the microbial community with abundance of genes in metabolism, cell-adhesion, motility, and quorum sensing. Moreover, Quaternary Ammonium Compound (QAC) resistance genes were also observed, this is significant as QAC sanitizers are commonly used in many food processing facilities. Multi-functional genes such as transposases, polymerases, permeases, flagellar proteins, and Mobile Genetic Elements (MGEs) were found suggesting these are dynamic microbial communities that work together to protect themselves against environmental stresses through multiple defense mechanisms.

**Conclusion:**

This study provides a framework for understanding the collective microbial network spanning a beef processing system. The results can be used to develop intervention strategies to best control these highly communicative microbial networks.

## Introduction

In the United States, an estimated 9 million people fall ill due to food-borne illness each year, resulting in 56,000 hospitalizations and 1,300 deaths (CDC, 2017). From 1998 to 2020, 1,287 outbreaks associated with food have occurred, out of which 960 were caused by *Salmonella*, 272 due to *E*.*coli* O157, and 55 due to *Listeria monocytogenes* (Interagency Food Safety Analytics Collaboration, 2022). Ensuring food safety is of utmost importance as the incidence of food-borne illnesses continues to rise. Microbial pathogens can thrive in biofilms and lead to foodborne illnesses, making biofilms a concern in food-processing environments (Srey et al., 2013; Dass and Wang, 2022). Biofilms are complex communities of microorganisms that adhere to surfaces and form a protective extracellular matrix, making them resistant to cleaning and disinfection (Van Houdt and Michiels, 2010; Yin et al., 2019). The metabolic potential of microorganisms within biofilms allows them to adapt to nutrient limitations and other environmental stressors, contributing to their persistence (Yin et al., 2019). This leads to the accumulation of pathogenic and spoilage microorganisms that may contaminate food products causing subsequent health and economic issues. Globally, the ability of microorganisms to form biofilms and pathogens to persist within them is a significant challenge for the food industry, underscoring the importance of understanding the underlying mechanisms and developing effective control measures.

To gain a deeper understanding of the complex microbial communities within biofilms in food-processing environments, researchers have increasingly turned to metagenomic approaches such as shotgun metagenomics and whole-genome sequencing (WGS). These methods have become popular because they enable the identification and characterization of the entire microbial community, including microorganisms that are difficult to culture or detect using traditional methods (Yadav et al., 2013). WGS data can predict the presence of genes and their functions within the community and can be used to reconstruct the metabolic pathways present. This entails identifying groups of genes that collaborate to carry out specific biochemical reactions and connecting these groups of genes (Tu et al., 2017). Comparative analysis of WGS data from different biofilm communities can provide insights into the factors impacting their metabolic capabilities. Metagenomic sequencing can potentially improve our understanding of biofilm formation and persistence and aid in the development of control measures. Several recent studies have utilized metagenomic approaches to characterize biofilms in various settings (Ding et al., 2019; Caraballo Guzmán et al., 2020; Rehman et al., 2020; Voglauer et al., 2022; Wang et al., 2022; Zhou et al., 2022; Zhu et al., 2022).

This research aimed to analyze biofilm composition, diversity, and functional potential in three beef-processing plants to further our understanding of the microbiome present in biofilms that can form in food-processing environments. Samples recovered from floor drains located in five distinct areas starting where finished carcasses exit the harvest floor (hot scale), then following the process flow to the hotbox where hot beef carcasses are rapidly chilled under intermittent sprays of cold water (hotbox). Further samples were collected from coolers where chilled carcasses are held and sorted (cooler), before entering fabrication or processing (processing). With the last location sampled being the grind room where trimmings left over from processing are made into ground beef (grind room). Two time points separated by 3 years were examined. By analyzing the microbial communities present in these samples, we gained insights into the factors that contributed to biofilm formation and persistence, which can inform the development of effective intervention strategies to control biofilm formation in meat-processing environments.

## Materials and methods

### Processing plant description

A total of 75 samples were collected from drains at three different beef-processing plants: Plant A (n=23), Plant B (n=23), and Plant C (n=29). The samples were collected at five locations within each plant, namely hotscale (n=4), hotbox (n=17), cooler (n=28), processing (n=15), and grind room (n=11). Samples were collected at two different time points, first in 2017 and 2018, and again in 2021. To keep the sampling distribution equal and avoid missing samples in one year, the time points 2017 and 2018 were combined and renamed T1. As a result, the final time points were set as T1 (2017-18) and T2 (2021). The beef processing plants were geographically separated, where Plants A and B were separated by approximately 200 miles, Plants B and C separated by approximately 400, and Plants A and C separated by approximately 600 miles.

### Sample collection, processing, and shotgun sequencing data acquisition

The samples were collected from floor drains as described previously using cellulose sponges wetted with buffered peptone water (Caraballo Guzmán et al., 2020). Sponges were hand massaged and 5 mL was removed and centrifuged (10,000 x G) to pellet cells. The pellet was resuspended in 750 µL bead suspension buffer from the Qiagen DNeasy Powerlyzer PowerSoil kit (Qiagen, Germantown MD), and homogenized in a Fastprep96 (MP Biomedicals, Irvine CA) bead beater (1,400 rpm 3 min). Homogenate was then split evenly into two 2 mL tubes and processed for DNA isolation according to the DNeasy kit insert.

Extracted DNA was quantified using a Qubit 4 Fluorometer (Thermo Fisher Scientific, Waltham, MA, USA). 100 ng of DNA from each sample were prepped for subsequent sequencing using an Illumina DNA Prep Kit (96 Samples) and Nextera™ DNA Indexes (96 Samples) (Illumina, San Diego, CA, USA) per manufacturer’s instructions. Prepped DNA was analyzed for fragment size distribution using an Agilent BIoAnalyzer 2100 and an Agilent High Sensitivity DNA Kit (Agilent Technologies, Santa Clara, CA, USA) and concentration was measured using the Qubit 4 Fluorometer.

Prepped DNA samples were pooled, denatured, diluted to appropriate concentration, and loaded into the MiSeq instrument (Illumina, San Diego, CA, USA) using an Illumina MiSeq Reagent Kit v3 (600 cycle) per manufacturer’s instructions. The paired-end sequencing run was set to 2x250 cycles. The FASTQ files generated were used for all downstream analyses.

### Sequence quality control, taxonomic assignment

An initial quality check was done using the program FastQC v0.11.9 (https://www.bioinformatics.babraham.ac.uk/projects/fastqc/) to determine the quality profile of the shotgun sequencing reads. Quality trimming was done using Trimmomatic v0.39 (Bolger et al., 2014) with a PHRED cutoff score of 20 and a minimum length of 50. Microbial reads were enriched by mapping the trimmed reads to the *Bos taurus* cattle genome (https://www.ncbi.nlm.nih.gov/assembly/GCF_002263795.2) using BWA-0.7.17 (r1188) aligner (Li and Durbin, 2009) and thereby removing the aligned reads. The filtered reads were used for taxonomic assignment using Kraken2 v.2.1.2 (Wood et al., 2019) by mapping against a custom Kraken database, Minikraken v1 (https://benlangmead.github.io/aws-indexes/k2), a pre-built Kraken database containing Refseq: bacteria, archaea, and viral sequences, was used for taxonomic assignment. Virsorter2 v.2.2.3 (Guo et al., 2021) was used to filter out the viral sequences in the assembly. Fungal reads were identified *via* a command-line BLAST search against the UNITE fungal database (https://unite.ut.ee/) with a percent similarity >80% and e-value of 1E^-5^.

### *De novo* assembly and metagenome binning

MEGAHIT v1.2.9 (Li et al., 2015) was used to perform *de novo* assembly with default parameters to obtain contigs. Pooled assembly was done by concatenating all the reads together to maximize the possibility of obtaining metagenome assembled genomes (MAGs) or bins. To perform metagenome binning, contigs >=1000bp were filtered using the program Seqtk 1.0-r31. Metagenome binning was done for the filtered contigs using the program MaxBin-2.2.7 (Wu et al., 2016) with default parameters and the quality of the obtained bins were checked using CheckM v.1.2.0 (Parks et al., 2015). High quality MAGs were selected based on the calculation “Quality = Completeness – 5 X Contamination” (Kim et al., 2022). Taxonomic assignments for the MAGs along with the phylogenetic classification were performed using Genome Taxonomy Database Toolkit v.2.1.1 (GTDB-tk) and Microbial Genome Atlas v.1.0.0 prima (MiGA).

### Functional annotation and pathway analysis for assemblies

Assemblies were generated separately for each plant for a particular time point and were given identifiers accordingly (Plant A-T1, PlantA-T2, and so on). Redundant contigs were removed from the assembly using CD-HIT v.4.8.1 program (Li and Godzik, 2006) with parameters (complementarity=99%, word size=8). For functional analysis, the minimum contig length was set to 500 bp. The length filtered contigs were uploaded to MG-RAST v.4.0.3 web server (Keegan et al., 2016) and eggNOG mapper v.2.1.8 (Cantalapiedra et al., 2021) to perform functional annotation. Pathway mapping was done based on databases: Kyoto Encyclopedia of Genes and Genomes KEGG Release 105.0 (Kanehisa et al., 2017) and SEED subsystems v.2.0 (Overbeek et al., 2014). CAZy database (Cantarel et al., 2009) was used for identifying the carbohydrate binding molecules.

### Mobile genetic elements, lateral gene transfer, and antimicrobial resistance gene identification

To identify genes associated with mobile genetic elements, lateral gene transfers (LGT) and antimicrobial resistance occurring among microbial communities the following tools were used. Mobile Genetic Elements (MGEs) were identified using the MOB-Suite program v.3.0.3 (Robertson and Nash, 2018). Potential LGT events were identified by the draft version of Workflow to Annotate Assemblies and Find LGT Events (WAAFLE) v.0.1.0, (https://huttenhower.sph.harvard.edu/waafle/). Lastly, AMRFinderPlus v3.11.4 by NCBI (Feldgarden et al., 2021) was used to identify antimicrobial resistance genes in the assemblies.

### Statistical analysis

Abundance tables were generated from Kraken2 reports using multiple scripts of KrakenTools v1.2 (Wood et al., 2019) (https://github.com/jenniferlu717/KrakenTools). Alpha and Beta diversity metrics were calculated using R package phyloseq v1.36.0 (McMurdie and Holmes, 2013). Core microbiome analysis was performed by obtaining the prevalence scores of taxa using the web server MicrobiomeAnalyst (Chong et al., 2020). Kruskal-wallis test was performed in R to test the significance of the alpha diversity metrics. Plots were generated using the R package ggplot2 v3.3.6 (Wickham, 2016). Heatmaps were generated using in-house R scripts. RStudio 2022.07.1 Build 554 was used in this study. All the R scripts used in this study are available in GitHub.

## Results

### Sample information, sequence data and assembly statistics

Shotgun metagenomic sequencing of the 75 biofilm samples yielded approximately 55.9 million 250 bp reads (or 13.9 billion bp) (**Supplementary Table S2**). There was an average number of 746K reads per sample. Quality control (QC) of the raw reads was performed using Trimmomatic with a minimum PHRED score of 20 and a minimum length of 50 bp, which removed 12.57% of the reads. Since the biofilm samples were collected from drains that contain microorganisms and run off from beef carcasses, blood, and tissues, it was appropriate to eliminate host bovine sequences for further analysis (Yang et al., 2016). The QC reads were then mapped onto the *Bos taurus* cattle genome, and the aligned reads were discarded. Finally, after removing host reads, 19.3 million potential microbial reads were retained for further analysis, providing an average of 258.2K per samples with a range from 8,290 to 759,974 reads.

Multiple *de novo* assemblies were performed, and samples were grouped based on specific plants and years. The number of contigs ranged from 41,598 (Plant B-T1) to 73,350 (Plant A-T2) with the contig N50 value ranging from 825 (Plant B-T1) to 1057 (Plant C-T2) (**Supplementary Table S1**).

### Taxonomic assignment and relative abundance of taxa

Kraken2 was used to assign taxonomic classifications to the potential microbial reads. Minikraken v1, a pre-built Kraken database containing Refseq: bacteria, archaea, and viral sequences, was used for taxonomic assignment. Since Kraken2 program is a read-based tool, *de novo* assembly was not required for taxonomic assignment. Kraken2 reports provided taxonomic information as well as abundance data for each taxon. The relative abundance was calculated using Kraken2 reports. Across the entire sample set, 40 phyla, 329 families, and 1034 genera were identified. Around 6.06% of the reads at the genus level were unclassified, ranging from 0.299% (plant B-T1) to 2.63% (plant C-T2).

**Figure 1:**
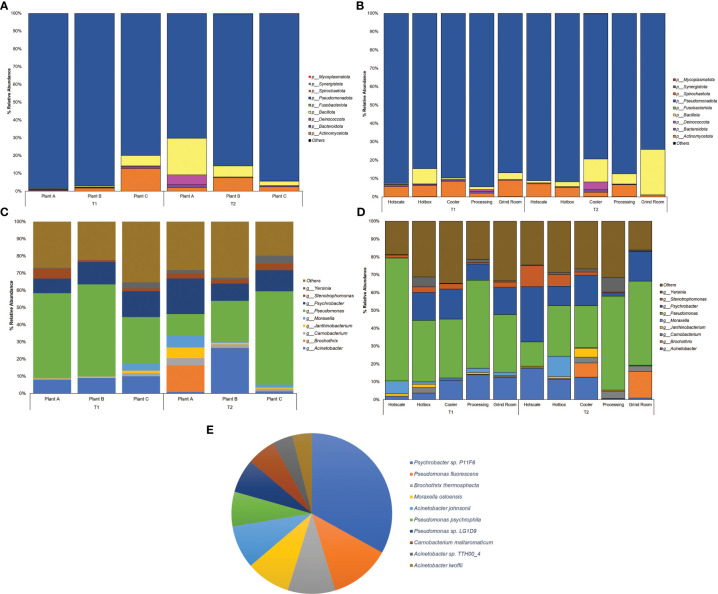
Percent relative abundance bar plots for Phylum of **(A)** Plants **(B)** Locations and genera of **(C)** Plants **(D)** Locations and **(E)** Pie Chart representing the abundance of species across the entire sample dataset.

Among the classified phyla, *Pseudomonadota* was observed to be the dominant phylum across all three plants, with the abundance ranging from 70.24% to 98.46% (**Figure 1A**) and locations with a range from 74.02% to 94.5% (**Figure 1B**). The highest abundance of *Pseudomonadota* was recorded in plant A, with an abundance of 98.46% in T1, followed by 97.06% in plant B-T1. In T2, plant C also observed a higher abundance of *Pseudomonadota* with 94.26%. *Pseudomonadota* dominated the abundance, leaving limited space for other phyla. The next most abundant phylum was *Bacillota*, which reached a maximum abundance of 20.55% in Plant A-T2. Rest of the samples had <10% abundance of *Bacillota*. The same was observed in the case of *Actinomycetota* in Plant C-T1 with the highest abundance of 12.66%. Location-wise, *Pseudomonadota* was found with >75% of abundance in all the locations with the highest being in processing with 94.4% abundance during T1 and hotbox with an abundance of 91.64% at T2. In the case of *Bacillota*, besides grind room-T2 (24.63%) and cooler-T2 (12.44%), the rest of the locations had <10% abundance. Highest abundance of *Actinomycetota* was 7.10% observed in hotscale-T2.

At the family level, *Pseudomonadaceae* was observed to be highly dominant family with abundances recorded in plant C-T2(25.54%), plant B (25.05%) and plant A (22.5%) in T2 (**Supplementary Figure S1A**). Location-wise hotscale in T1 had the highest abundance of *Pseudomonadaceae* (31.76%) followed by processing (22.53%) and hotbox (15.99%) (**Supplementary Figure S1B**). In T2, processing had the highest abundance of *Pseudomonadaceae* (23.91%) followed by grind room (22.8%) and hotbox (13.45%). *Moraxellaceae*, the second most abundant family was observed in hotscale (22.52%) in T2 followed by hotbox (15.44%) and cooler (14.07%). In T1, grind room had the highest *Moraxellaceae* abundance (13.25%) followed by cooler (12.65%) and processing (11.24%). Considering the plants, plant B had the highest abundance of *Moraxellaceae* (16.90%) in T2 followed by plant A (13.27%). In the year T1, plant C showed an abundance of 12.78% of *Moraxellaceae*.

At the genus level, *Pseudomonas* showed the highest dominance in plant B-T1 recording the highest abundance (15.42%) followed by plant A (14.07%) (**Figure 1C**). In T2, plant C showed the highest abundance (15.95%) followed by plant B (7.06%). Location-wise, hotscale had the highest abundance of *Pseudomonas* (19.49%) in T1 followed by processing (13.82%). However, in T2, processing had an abundance of 14.77% of *Pseudomonas* followed by grind room (14.59%). *Acinetobacter* was the second most abundant genus amongst the plants, with an abundance of 7.76% in plant B-T2. Considering locations, the highest abundance of *Acinetobacter* was recorded in hotscale with an abundance of 5.14% in T2 followed by cooler (3.8%) and hotbox (3.35%) in the same year (**Figure 1D**). In T1, processing showed a 3.91% abundance of *Acinetobacter* in processing and a 3.55% abundance in grind room. *Psychrobacter* was the next most abundant genus observed, with the highest abundance in plant A-T2 (6.29%) followed by plant C-T2, showing a 3.61% abundance. In T1, plant C had a *Psychrobacter* abundance of 4.22% followed by plant B (3.75%).

### Microbial diversity in the drain biofilms of beef-processing plants

**Figure 2:**
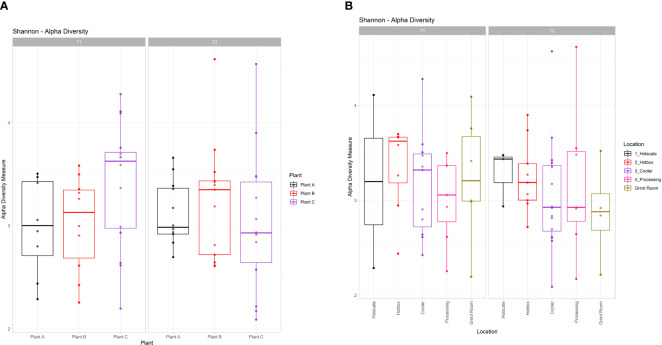
Box and whiskers plots showing the alpha diversity calculated through Shannon index for **(A)** Plants and **(B)** Locations in T1 and T2. Boxes indicate the interquartile range (IQR) with the dashed line inside the box representing the median value of alpha diversity.

Alpha diversity analysis was performed for both plants and locations in T1 and T2 (**Figures 2A, B**). Overall, there was no significant difference in alpha diversity based on the Shannon metric between plants (Kruskal-Wallis test; χ^2 ^= 6.1791; p=0.2892) or locations (Kruskal-Wallis test; χ^2 ^= 2.0876; p=0.8369). In T1 samples, the diversity pattern based on the median value of the Inter Quartile Range (IQR), showed an increase in diversity from hotscale to hotbox, followed by a decrease in the cooler and processing. Finally, an increase in the final location grind room. However, in T2, an overall decreasing profile from hotscale to grind room was observed. With the exception of hotscale, alpha diversity decreased for all locations between T1 and T2. Between T1 and T2, the microbiota of beef-processing plants showed contrasting trends in alpha diversity. In T1, the diversity increased from plant A to plant C, with plant B showing a slight decline. However, the opposite trend was observed in T2, with a decrease in diversity from plant A to plant C and an increase in plant B. Diversity in plant A remained nearly constant between T1 and T2, while diversity in plant B increased and diversity in plant C decreased. Principal coordinate analysis (PCoA) was used to perform beta diversity analysis based on the unweighted unifrac metric. PCoA plots of the three beef-processing plants across two time points showed no visual clustering of samples, implying no diversity difference across the samples (**Supplementary Figures S2A-D**). PERMANOVA analysis confirmed the above by showing no significant difference in beta diversity across plant samples (F=1.2837; p=0.091). Similarly, for locations, no clustering was observed among the samples which was confirmed by PERMANOVA analysis (F=1.1939; p=0.159) showing no significant difference in beta diversity among the locations across the two time points.

### Core microbiome analysis among the beef-processing plants and locations

**Figure 3:**
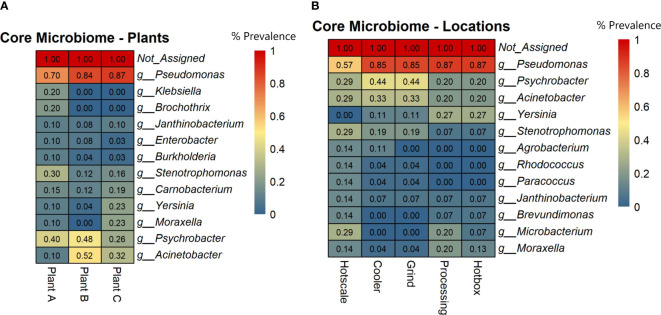
Heatmaps showing the core microbial genera across **(A)** Plants and **(B)** Locations based on the prevalence scores. Prevalence Score = the proportion of samples in this group with this taxon detected.

To identify highly prevalent taxa across all samples, a core microbiome analysis using MicrobiomeAnalyst with default parameters was performed (Sample Prevalence=>20%; Relative abundance=>0.01%). Prevalence scores were obtained for each taxon based on the plant and sample location and heatmaps were generated. The results showed that *Pseudomonas* was the most prevalent genus in all three plants, ranging from 70% in plant A to 87% in plant C (**Figure 3A**). *Psychrobacter* was the next most prevalent genus, with the highest prevalence in plants B (48%) and A (40%). *Acinetobacter* was prevalent in plants B (52%) and C (32%), but less prevalent in plant A (10%). *Stenotrophomonas* and *Carnobacterium* had a prevalence ranging from 12% to 30% across all plants. *Yersinia* and *Moraxella* were only prevalent in plant C, with 23% prevalence. The core microbiome analysis also revealed the most prevalent genera in different locations of the three beef-processing plants. *Pseudomonas* was the most common genus in the hotbox and processing (87%), followed by cooler and grind room (85%) and hotscale (57%) (**Figure 3B**). *Psychrobacter* was prevalent in cooler and grind room (44%) and hotscale (29%). *Acinetobacter* was found in cooler and grind room (33%) and hotscale (29%). *Yersinia* was prevalent in plant C processing, hotbox (27%), and cooler and grind room (11%). *Moraxella* was prevalent in Plant C processing (20%), followed by hotscale (14%) and hotbox (13%).

### Metagenome bins of pooled *de novo* assembly

To perform metagenome binning, contigs that were >1000bp were filtered out, and all of the individual assemblies were pooled together to form a single pooled assembly. The resulting assembly was used to perform metagenome binning using the program MaxBin2. In total, 49 bins were generated, and good quality bins were selected based on the parameters mentioned above. One of the bins (Bin name: Pooled-Bin-007) was identified as *Thermus parvatiensis* belonging to the phylum *Deinococcota* having a completeness of 99.79% with a contamination of 3.6% and an overall quality of 81.79%. Functional analysis of this bin revealed 2,668 coding DNA sequences (CDS) with 1,946 proteins having functional assignments and 722 hypothetical proteins. Phylogenetic analysis revealed the close relationship of this bacteria with *Thermus thermophilus* and *Thermus islandicus* (**Supplementary Figure S3A**). The second bin (Bin name: Pooled-Bin-021) was identified as *Hydrogenophilus thermoluteolus* also known as *Pseudomonas hydrogenothermophila* belonging to the phylum *Pseudomonadota*. The second bin had a completeness of 86.93% with a contamination of 3.84% and an overall quality of 67.73%. Functional analysis showed 2,286 CDS with 1,536 proteins with functional assignments and 750 hypothetical proteins. Phylogenetic analysis showed a close relationship of this bacteria with *Methyloversatilis* sp. and *Burkholderia vietnamiensis* (**Supplementary Figure S3B**).

### Functional potential of microbiome in biofilms

**Figure 4:**
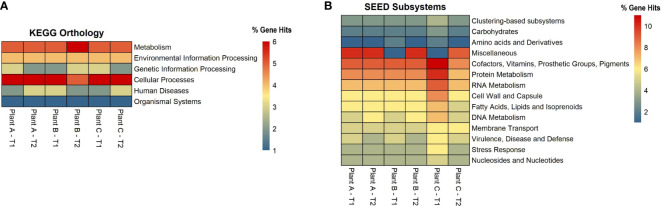
Heatmaps showing the number of gene hits related to diverse pathways based on the annotation using **(A)** KEGG and **(B)** SEED Subsystems databases.

For each assembly, functional analysis was performed, the eggNOG mapper was used to obtain protein annotations for each contig, and pathways were identified using the web-server MG-RAST, which is based on the KEGG Orthology and SEED subsystems databases (**Figures 4A, B**). According to the KEGG pathway analysis, the microbiome had a high metabolic potential observed in all samples, with 55% of genes involved in metabolism. Plant A and B had 57% of the metabolism genes in T1, followed by 56% in plant A-T2, plant B-T1, and plant C-T1. More than or equal to 20% of the genes in all samples were discovered to be involved in Environmental Information Processing, which includes pathways for membrane transport, signal transduction, and signaling molecules and interaction. For genetic information processing, which includes molecular processes such as transcription, translation, replication, repair, folding, sorting, and degradation pathways, gene hits ranged from 14% to 17%. 5% to 7% of the genes were involved in cellular processes *viz*., transport and catabolism, cell growth and death and cell motility.

Based on the SEED subsystems database, ≥16% of the genes were involved in clustering-based systems which includes proteosomes, ribosomes and recombinant-related clusters (Delmont et al., 2012). Around 13 to 15% of the genes were found to be involved in carbohydrates metabolism which was around the same (12-13%) as amino acids and derivatives biosynthesis. Other metabolism related pathways such as proteins, RNA and DNA metabolism had 4-7% of the gene hits present in them. Around 3-4% of the genes were discovered to be involved in stress response and virulence, disease, and defense. 3-5% of the gene hits were observed in membrane transport. Numerous proteins associated with potential biofilm formation were detected in the drains of the beef-processing plants, including various cell attachment proteins such as Adhesins (most abundant in plant B-T2; n=100). Proteins facilitating motility, such as Flagellins (highly present in plant A-T2; n=97), Motility Protein A (abundant in plant C-T2; n=16), Motility Protein B (prominent in plant C-T2; n=27), and pilins (found in large quantities in plant A-T2; n=46), were also identified. In addition to these, a few genes related to polysaccharide and alginate biosynthesis were discovered, namely PslA, Pel, and Alg44, which contribute to the integrity of the EPS matrix. The presence of transposases was also noted in significant abundance (n=>800) across all samples, with the highest levels observed in Plant A-T2 (n=1573).

Potential lateral gene transfer events for each assembly were identified using the WAAFLE tool. While the assembly of plant B-T1 showed no LGT events, the other assemblies showed >50 LGT events (**Table 1**), possibly due to its being a new tool still in development. The highest number of LGT events were recorded for plantA-T2 followed by plant C-T1 (67), plant B-T2 (63), plant C-T2 (52), and finally, Plant A-T1 (51). The tool attempts to predict the synteny of the transfer event, explaining the gene contribution from each of two clades. The synteny of the transfer events was mostly identified as AB with contributions from clade A and clade B. The tool also attempts to provide the putative direction of the gene transfer event with B>A, meaning that A genes flank the inserted gene B, and A?B indicating that the direction is uncertain. Mostly, the direction of the transfer events was uncertain (A?B), with only 9 B>A events identified. Antimicrobial resistance genes (ARG), were identified by the tool NCBI-AMRfinderPlus and the pooled assembly of the entire datasets were used as input for this analysis. This analysis identified 24 ARGs across the pooled assembly, including diverse classes of antimicrobials and sanitizers such as quaternary ammonium compounds (QAC; **Supplementary Table S5**). Mobile genetic elements were identified using the software MOB-Suite. For this, the entire pooled assembly of all samples clustered together was used as input. From the results, 299 circular molecules of both chromosomal and plasmid origin were identified across the entire pooled assembly, demonstrating the microbial communities in the biofilm possess the ability to actively transfer genetic information.

**Table 1:** Number of putative lateral gene transfer (LGT) events identified across all the different assemblies.

Sample	Number of putative LGT events
Plant A – T1	51
Plant A – T2	74
Plant B – T1	0
Plant B – T2	63
Plant C – T1	67
Plant C – T2	52

### Phages and fungi detected in the beef-processing environment

The program Virsorter2 was used to identify the viral sequences in the pooled assembly. The results showed the presence of 355 phage contigs in the assembly (**Supplementary Table S3**). A high abundance of *Pseudomonas* phages (n=233) was observed across the pooled assembly along with other phages for *Escherichia, Aeromonas, Xanthomonas*, and other genera. Similarly, command-line BLAST analysis using the UNITE database for Fungi yielded only 19 contigs belonging to Fungi in the pooled assembly (**Supplementary Table S4**). Fungal genera identified were *Rozellomycota, Tausonia, Ascomycota, Fuscoporia* etc.

## Discussion

Biofilms are a major challenge to food-processing environments because they may harbor food spoilage organisms and foodborne pathogens. Multi-species biofilms, which are commonly found in food-processing environments, may create an ecological niche for pathogens for better survival by conferring resistance to sanitization (Donlan, 2001; Dass and Wang, 2022). Therefore, understanding the microbial diversity present in these multi-species biofilms and assessing their functional potential can provide valuable information for developing effective biofilm control strategies. Biofilm investigation studies have been conducted in the past, but are rare and limited to only specific environments (Voglauer et al., 2022). Metagenomic characterization of biofilms have already been documented in studies in a variety of settings, such as food-processing plants (Caraballo Guzmán et al., 2020; Wang et al., 2022), water-hoses (Voglauer et al., 2022), heritage stone surfaces (Skipper et al., 2022), bioreactors (Rehman et al., 2020; Zhou et al., 2022; Zhu et al., 2022) and in marine environment (Ding et al., 2019). However, there is a paucity of data regarding the biofilms in beef-processing plants.

This study analyzed drain samples from five different locations in three beef-processing plants at two different time points to determine their metagenomic profiles and microbial diversity. Microbial diversity profiles were determined based on both alpha and beta diversity analysis. Shannon index (Alpha diversity) accounts for both the species richness and evenness of a sample. Shannon index values ranged from 2.08 to 4.62 across all the samples and there were no radical differences in alpha diversity among both the plants and locations (p>0.05) for the two time points T1 and T2. Further, beta diversity analysis was based on Unweighted Unifrac metric. This metric accounts for the incidence of the micro-organism excluding the abundance of the same and detecting the rare lineages (Chen et al., 2012). The PCoA plots of beta diversity did not show any distinct group clustering, which was supported by the PERMANOVA analysis, which did not yield statistically significant results among groups (p>0.05). As a result, this indicates that similar microbiomes may exist in all three beef-processing plants, regardless of how far apart they are in space and time. However, the abundance of bacteria varied, with *Pseudomonas*, *Psychrobacter*, and *Acinetobacter* being common in all three plants but with little variation in their abundance. Based on the core microbiome analysis, these three genera were found to be the core microbiome of beef-processing plants.

The most ubiquitous genus was *Pseudomonas* which belongs to the phylum *Pseudomonadota*, a Gram-negative, chemoorganotrophic, rod-shaped bacteria (Iglewski, 1996). It was prevalent across all locations with a prevalence of ≥85% except for hotscale (57%). *Pseudomonas* is a versatile genus with 191 described species. This study found over 137 different *Pseudomonas* species in all samples. It has simple nutritional requirements and can use a wide range of organic compounds as carbon and energy sources and colonize a wide range of niches (Madigan et al., 2011). The study also found three dominant *Pseudomonas* species, including *P. fluorescens*, *P. psychrophila*, and an unclassified *Pseudomonas* sp. LG1D9. (**Figure 1E**). *P.fluorescens* is an obligate aerobe that can use nitrate instead of oxygen as a final electron acceptor during cellular respiration (Muriel et al., 2015). It has been found to cause food-spoilage, and some strains have the plasticity to grow in higher temperatures. The species is also known to form biofilms in various environments (O’Toole and Kolter, 1998; Bianciotto et al., 2001; Baum et al., 2009) and has been found in meat and dairy-processing environments (Stellato et al., 2017). Another *Pseudomonas* species found in the samples was *P. psychrophila*. These are psychrophilic bacteria that can grow in temperatures ranging from -20°C to 20°C (Abraham et al., 2020); it was present in all the locations, with higher abundance in the hotbox, where sample collection temperatures had ranged from 2.9°C to 11.4°C, thus supporting the psychrophilic characteristic of the species. One study has looked into the biofilm-forming ability of *P. psychrophila* and discovered that the synthesis of the quorum sensing signaling molecule C4-HSL doubled the biofilm-forming ability of the bacterium by increasing its attachment (Bai and Rai Vittal, 2014). In our study, functional annotation showed protein hits for acyl homoserine lactone synthase (AHL), which directs the synthesis of C4-HSL, which in turn aids in swarming motility in the quorum sensing pathway.

*Psychrobacter* is a gram-negative, psychrotrophic, osmotolerant and aerobic bacteria belonging to the family *Moraxellaceae*. In this study, it was found to be the second most abundant genus in all plants and locations except the hotscale in T1. However, there was an increase in its abundance in the hotscale in T2. Species-level identification showed the presence of an unclassified *Psychrobacter* sp. P11F6 that was abundant in the cooler of plant A, along with 12 other species that were not dominant in the samples. *Psychrobacter* can grow on both complex and simple media, with ammonium as a nitrogen source. It has a wide ecological distribution, from animal bodies to non-host environments, and is mostly associated with colder and saline domains. (Welter et al., 2021). The high abundance of *Psychrobacter* in the cooler might be related to the temperature range of the local sample collection site in the coolers of the three plants, which were between 2.9°C and 13.3°C at the time of collection. Some species of Psychrobacter are capable of movement by twitching motility (García-López et al., 2014). Unlike *Pseudomonas*, *Psychrobacter* does not readily form biofilms, and there is little evidence to support this. However, one species (*P.arcticus*) was found to be capable of forming biofilms with the help of an adhesin protein in minimal medium under laboratory conditions, when supplied with acetate as the sole carbon source with 1-7% sea salt at temperatures ranging from 4°- 22°C (Hinsa-Leasure et al., 2013). *P. arcticus* was found in low abundance in majority of the samples in this study, with the highest abundance recorded on a hotscale in plant A in T2. Some species of *Psychrobacter* produce anti-biofilm agents that disrupt the biofilm formation of other bacterial species. For example, *Psychrobacter* sp. TAE2020 produces *CATASAN*, a protein-polysaccharide complex that inhibits the ability of *Staphylococcus epidermidis* to form biofilms (D’Angelo et al., 2022). Similarly, another *Psychrobacter* species isolated from a marine sediment produced AHL-lactonase, which degrades the quorum sensing signaling molecule N-acylhomoserine lactones and thus controlled biofilm formation by *Pseudomonas* (Packiavathy et al., 2021).

*Acinetobacter*, a Gram-negative genus of aerobic, non-fermentative coccobacilli belonging to the *Gammaproteobacteria* class, was the third most prevalent genus found in the drains of the three beef-processing plants. A total of 33 different *Acinetobacter* species were found in all samples, with *A. johnsonii*, *A. lwoffii*, and an unclassified *Acinetobacter* sp. TTH00_4 being the most dominant. *A.baumannii* was abundant in the cooler of plant B in T1 but decreased in T2, and was also found in hotscale and hotbox. Some *Acinetobacter* species, primarily *A.baumannii* and *A. calcoaceticus*, have been found to form biofilms, and produce biofilm associated protein (BAP) that aids in the formation of biofilms and cell adhesion (De Gregorio et al., 2015). In one study, *A. calcoaceticus* improved the biofilm formation ability of Shiga toxin producing *E.coli* (STEC) in a meat-processing environment by increasing the surface colonization of the latter (Habimana et al., 2010).

Other genera found in various plants and locations include *Moraxella*, *Yersinia*, and *Stenotrophomonas*. *Moraxella osloensis*, a Gram-negative bacterium, was highly abundant in the hotbox of plant C in T2, while *Stenotrophomonas* was particularly abundant in the hotscale in T2. A biofilm formation study in drinking water distribution system pipes exposed to long-term high chlorine levels identified *M.osloensis* and *Stenotrophomonas* sp. biofilm community structure in all pipe materials (Zhu et al., 2014). *Yersinia* was slightly more abundant in the hotbox in T1 and processing in T2. These bacteria may participate in biofilm formation and coexist in the beef-processing environment through synergistic interactions with one another.

*Thermus parvatiensis*, found through metagenome binning, belonged to the phylum *Deinococcota*. The phylum *Deinococcota* was particularly abundant in plant A in T2. It can withstand temperatures ranging from 41° to 122°C and was previously isolated from a hot water spring located atop the Himalayan ranges in India (Dwivedi et al., 2015). Interestingly, in this study, it was found to be present in coolers with temperatures ranging from 2.9° to 13.3°C. Another microorganism identified through binning was *Hydrogenophilus thermoluteolus*, which was also abundant in the cooler of plant A. *H. thermoluteolus* was also moderately thermophilic, but evidence suggests that it was isolated from colder environments. DNA from *H.thermoluteolus* has been discovered in accretion ice in Antarctica’s subglacial Lake Vostok (Lavire et al., 2006).

Biofilm formation is a complex process involving the synthesis and secretion of a wide range of proteins by the microorganisms that comprise the biofilm. These microorganisms, including bacteria, fungi, and others, produce extracellular polymeric substances (EPS) containing polysaccharides, proteins, lipids, and nucleic acids, which are crucial for the structural integrity and adhesion of the biofilms (Limoli et al., 2015; Costa et al., 2018). Attachment is a crucial step in the colonization of an environment or host by bacteria. Adhesins are commonly found on bacterial pili, which are hair-like projections on the surface of bacterial cells. Adhesins may also be found on bacterial cell walls or membranes and are distributed differently depending on the type of bacteria, mediating the attachment of microorganisms to surfaces or neighboring cells (Kline et al., 2009). This study found that all the *de novo* assemblies contained numerous adhesins, with plant B having the most in T2. Previous studies have shown that adhesins play an important role in biofilm formation in bacteria (Pratt and Kolter, 1999; Nobile et al., 2006; Absalon et al., 2011).

Bacterial motility is also a crucial step in biofilm formation as it facilitates surface exploration, cell-to-cell communication, nutrient acquisition, and biofilm expansion. Flagella, a complex apparatus made up of 20 different proteins; and pili (also known as fimbriae): proteinaceous, filamentous polymeric organelles, both aid in bacterial motility, particularly type IV pili. One of the most important flagellar proteins is flagellin, a polymeric protein that makes up most of a bacterial flagellum and is located in the hollow cylinder of the flagellum with a helical structure. Motility proteins A and B, encoded by the genes *motA* and *motB*, respectively, are integral membrane proteins that form components of the flagellar motor. These proteins harness energy from forming pores and help the mechanical movement of flagella across the membrane through proton motive force (PMF) (Minamino et al., 2011). This study found abundant flagellin proteins across the three plants in T1 and T2, with the most abundant in plant A in T2. Although not as numerous as flagellin, there were fewer gene hits for motA and motB across the three plants in two time points*. FleQ*, a gene required for flagellar assembly in the cAMP/Vfr signalling pathway, had fewer gene hits in our datasets, with a maximum of 10 hits recorded for plant A in T1 and plant C in T2. Pilins are fibrous proteins that are found in the pilus of bacteria. Type IV pilins are structural modules that help with motility, attachment, electrical conductance, and DNA acquisition (Giltner et al., 2012). This study found several gene hits for pilins, with plant B in T2 having the most hits. The presence of these proteins suggests that bacteria are actively moving around the biofilm architecture, acquiring nutrients, and communicating with other bacteria.

A complex network of genes and proteins governs polysaccharide biosynthesis in biofilms. *Psl* genes (*PslA*) and Pel genes (*PelA, PelB, PelC, PelD, PelE, PelF*, and *PelG*) play critical roles in polysaccharide biosynthesis in the c-di-GMP signalling pathway (Colvin et al., 2012). The gene *Alg44* (mannuronan synthase) is involved in the biosynthesis of alginate (Franklin et al., 2011). However, these genes were present in relatively low numbers in most of the samples, with the exception of plant A in T2, which had no hits for both the *Psl* and *Pel* genes.

Enzymes are also important in forming biofilms, particularly glycosyl transferases (GTs), which are responsible for synthesizing exopolysaccharides (EPS) that make up the biofilm matrix (Wang et al., 2020). These enzymes transfer sugar molecules from activated nucleotide sugars to growing polysaccharide chains, helping to maintain the structural stability of biofilms. Several studies have shown that inactivating or deleting the GTs reduced biofilm formation (Tao et al., 2010; Theilacker et al., 2011; Rainey et al., 2018) emphasizing the importance of these enzymes. In this study, GTs were identified using the CAZy database (Cantarel et al., 2009), and several hits were found for GTs in all the assemblies, with the highest being 736 hits in plant B in T2.

An interesting discovery from the functional analysis is that, despite observing differences in alpha diversity and varying microbial signatures across the datasets, the functional potential remained consistent (**Figure 4**). This phenomenon can be explained by an ecological property known as functional redundancy (Tian et al., 2020). Numerous studies across different niches, such as the gut microbiome, macroalgae, and plants, have demonstrated that while the taxonomic composition of microbiomes varies significantly, the functional genes remain conserved (Turnbaugh et al., 2008; Burke et al., 2011; Louca et al., 2016). The persistence of functional redundancy, even following changes in the microbiome, may be attributed to the emergent properties of microbial systems resulting from biotic interactions, as well as other environmental and spatial processes (Louca et al., 2018).

**Figure 5:**
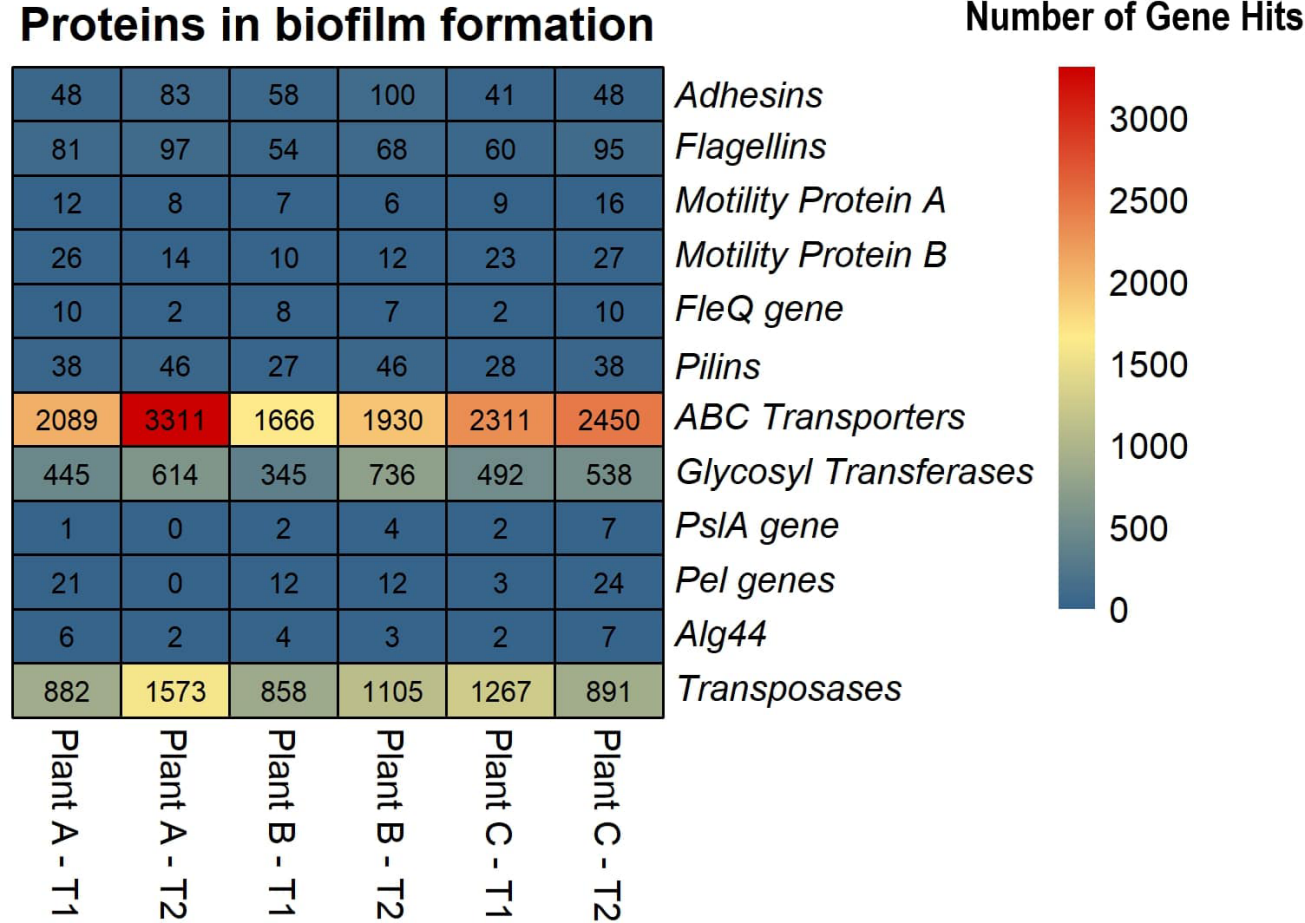
Heatmap displaying the abundance of selected proteins in biofilm formation across the beef-processing plants for two time points T1 and T2.

Lateral gene transfer, also referred to as horizontal gene transfer, is a significant driver of microbial evolution and niche adaptation (Song et al., 2021). Lateral gene transfer and biofilms are linked processes because biofilms provide plasmid stability, which enhances the transfer of mobile genetic elements (Madsen et al., 2012). Predicting the presence of LGT events in the genome of an organism is a direct analysis, but identifying LGT events in a sequence data from a community is a challenge. The study did not detect any LGT events for plant B in T1, while they were putatively present in the other assemblies. The highest LGT events were observed in plant A in T2, with a functional annotation of the transferrable genes revealing a diversity that included those related to flagellar proteins, transposases, polymerase, kinases, integrases, and ABC transporters. ABC transporters are integral membrane proteins that play a crucial role in transporting substrates across membranes with the aid of ATP (Rees et al., 2009). They are essential in the transition from reversible to irreversible attachment during biofilm formation (Hinsa et al., 2003) and also helps cope with desiccation tolerance. Transposases are enzymes that recognize and bind to the inverse terminal repeats in DNA, thereby cleaving DNA transposons (Hickman and Dyda, 2015). These enzymes facilitate the movement of DNA segments between nonhomologous sites, which plays a critical role in lateral gene transfer (Craig, 2002; Craig et al., 2002). The presence of transposases in biofilms, particularly in high abundance, suggests that the communities are primed to transfer genetic material and adapt to changing conditions. Previous studies have reported 3,735 transposases in biofilm formed in hydrothermal chimneys covering more than 8% of the metagenomic reads (Brazelton and Baross, 2009). Higher abundance of transposases were observed in Zn-associated marine biofilms (Ding et al., 2019), acid mine drainage biofilms (Tyson et al., 2004), and upregulated transposases in *Treponema denticola*, a bacterium that exists as part of a dense biofilm in subgingival dental plaque accumulated in teeth (Mitchell et al., 2010). This study did not find those levels, with only more than 800 transposases identified in all samples, with the highest number observed in plant A-T2 (n=1573) (**Figure 5**).

Antimicrobial resistance genes (ARGs) were minimally detected in beef-processing plants, and while this study detected a number of different antimicrobial resistance genes being present in the drains, (**Supplementary Table S5**). The most notable were quaternary ammonium compound (QAC) resistance genes. QACs are cationic surfactants used in disinfectants and biocide. These compounds are widely used as sanitizers in the food processing industry. The structure of QAC consists of a quaternary ammonium nitrogen linked by four alkyl or aryl groups and an anionic ion like chloride or bromide. One of the alkyl groups is a long hydrophobic chain containing more than eight hydrocarbons, which provides antimicrobial properties to QACs (Sun, 2011). This study revealed the presence of five QAC resistance genes (*QacL, QacE, Qac-pB8, QacF*, and *QacG2*). A previous study has shown that exposure to QAC (cetyltrimethylammonium bromide (CTAB)) can enhance the expression of antimicrobial resistance genes in O_2_-based membrane biofilm reactors (O_2_-MBfRs) (Luo et al., 2021). Therefore, the detection of QAC resistance genes in the drains of beef-processing plants suggests that the microbial communities present in these environments may have the potential to develop resistance to QACs. The prevalence of QAC resistance in biofilms is therefore not surprising.

## Conclusion

This study demonstrates the microbial diversity and functional potential of the microbiome in a beef-processing environment. Our findings indicate a high level of microbial alpha diversity across all three beef-processing plants. The study also identified the necessary protein arsenal possessed by microbial assemblages, enabling them to withstand environmental stresses. This study provides a comprehensive snapshot of the microbial profile of biofilms found in the meat-processing environment. To gain a better understanding of the dynamic microbial community in beef-processing environmental biofilms, transcriptomic studies should be conducted in the future, and focus on identifying gene expression profiles in real-time, providing a more comprehensive understanding of the behavior of microbial communities.

## Data availability statement

The data analyzed in this study is subject to the following licenses/restrictions: The authors declare that the data supporting the findings of this study are presented within the article and the **supplementary information files**. The fastq.qz files of the Illumina paired-end sequencing and metadata originate from meat processing plants, and authors are obliged to maintain confidentiality, preventing the public deposition of these sequences. However, in case of a reasonable request, the authors are fully capable and willing to make these data available through file sharing. The data was generated under a non-disclosure agreement with meat processors, and we can share data with those willing to be bound by the same non-disclosure agreement. Requests to access these datasets should be directed to the corresponding author. https://github.com/dasslab/ShotgunTAMUData.

## Author contributions

VP, JB, and SD contributed to the conception and design of the study. VP, DB and SV performed the metagenomic analysis and VP wrote the first draft of the manuscript. JB and SD revised the manuscript critically for important intellectual content. All authors contributed to the article and approved the submitted version

## Supplementary material

Supplementary Figure S1 Percent Relative Abundance bar plots showing the abundance at the family level for **(A)** Plants and **(B)** Locations.

Supplementary Figure S2 PCoA plots displaying the beta diversity based on Unweighted Unifrac metric for the plants in **(A)** T1 **(B)** T2 and for the locations in **(C)** T1 and **(D)** T2. Different colored ellipses represent the spread or variability of the data points within each group. Larger ellipses indicate higher dispersion, meaning greater variation in the sample while smaller ellipses indicate more tightly clustered samples with lesser dispersion.

Supplementary Figure S3 Phylogenetic trees for metagenome bins of **(A)** *Thermus parvatiensis* and **(B)** *Hydrogenophilus thermoluteolus*.

Supplementary Table S1 Assembly Statistics.xlsx.

Supplementary Table S2 Sequence statistics and metadata for all drain biofilm samples.xlsx.

Supplementary Table S3 Viral Hits in Pooled Assembly.xlsx.

Supplementary Table S4 Fungal hits in Pooled assembly.xlsx.

Supplementary Table S5 Antibiotic Resistance Genes (ARGs) observed across the pooled assembly.xlsx.

## References

[B1] Abraham W. P. Raghunandanan S. Gopinath V. Suryaletha K. Thomas S. (2020). Deciphering the Cold Adaptive Mechanisms in Pseudomonas psychrophila MTCC12324 Isolated from the Arctic at 79°N. Curr. Microbiol. 77, 2345–2355. doi: 10.1007/S00284-020-02006-2/FIGURES/3 32363422

[B2] Absalon C. Van Dellen K. Watnick P. I. (2011). A communal bacterial adhesin anchors biofilm and bystander cells to surfaces. PloS Pathog. 7, e1002210. doi: 10.1371/JOURNAL.PPAT.1002210 21901100 PMC3161981

[B3] Bai A. J. Rai Vittal R. (2014). Quorum sensing regulation and inhibition of exoenzyme production and biofilm formation in the food spoilage bacteria pseudomonas psychrophila PSPF19. Food Biotechnology 28, 293–308. doi: 10.1080/08905436.2014.963601

[B4] Baum M. M. Kainović A. O’Keeffe T. Pandita R. McDonald K. Wu S.. (2009). Characterization of structures in biofilms formed by a Pseudomonas fluorescens isolated from soil. BMC Microbiol. 9, 103. doi: 10.1186/1471-2180-9-103 19460161 PMC2697165

[B5] Bianciotto V. Andreotti S. Balestrini R. Bonfante P. Perotto S. (2001). Mucoid mutants of the biocontrol strain pseudomonas fluorescens CHA0 show increased ability in biofilm formation on mycorrhizal and nonmycorrhizal carrot roots. Mol. Plant Microbe Interact. 14, 255–260. doi: 10.1094/MPMI.2001.14.2.255 11204790

[B6] Bolger A. M. Lohse M. Usadel B. (2014). Trimmomatic: A flexible trimmer for Illumina sequence data. Bioinformatics 30, 2114–2120. doi: 10.1093/bioinformatics/btu170 24695404 PMC4103590

[B7] Brazelton W. J. Baross J. A. (2009). Abundant transposases encoded by the metagenome of a hydrothermal chimney biofilm. ISME. J. 312 3, 1420–1424. doi: 10.1038/ismej.2009.79 19571895

[B8] Burke C. Steinberg P. Rusch D. Kjelleberg S. Thomas T. (2011). Bacterial community assembly based on functional genes rather than species. Proc. Natl. Acad. Sci. United. States America 108 (34), 14288–14293. doi: 10.1073/PNAS.1101591108/SUPPL_FILE/PNAS.201101591SI.PDF PMC3161577 21825123

[B9] Cantalapiedra C. P. Herņandez-Plaza A. Letunic I. Bork P. Huerta-Cepas J. (2021). eggNOG-mapper v2: Functional Annotation, Orthology Assignments, and Domain Prediction at the Metagenomic Scale. Mol. Biol. Evol. 38, 5825–5829. doi: 10.1093/MOLBEV/MSAB293 34597405 PMC8662613

[B10] Cantarel B. I. Coutinho P. M. Rancurel C. Bernard T. Lombard V. Henrissat B. (2009). The Carbohydrate-Active EnZymes database (CAZy): an expert resource for Glycogenomics. Nucleic Acids Res. 37, D233. doi: 10.1093/NAR/GKN663 18838391 PMC2686590

[B11] Caraballo Guzmán A. González Hurtado M. I. Cuesta-Astroz Y. Torres G. (2020). Metagenomic characterization of bacterial biofilm in four food processing plants in Colombia. Braz. J. Microbiol. 51, 1259–1267. doi: 10.1007/s42770-020-00260-x 32221908 PMC7455661

[B12] CDC (2017). Surveillance for foodborne disease outbreaks United States 2015: annual report. CDC. Atlanta. G 62 (1), 91–93. doi: 10.1016/j.annemergmed.2013.04.001

[B13] Chen J. Bittinger K. Charlson E. S. Hoffmann C. Lewis J. Wu G. D.. (2012). Associating microbiome composition with environmental covariates using generalized UniFrac distances. Bioinformatics 28, 2106–2113. doi: 10.1093/BIOINFORMATICS/BTS342 22711789 PMC3413390

[B14] Chong J. Liu P. Zhou G. Xia J. (2020). Using MicrobiomeAnalyst for comprehensive statistical, functional, and meta-analysis of microbiome data. Nat. Protoc. 15, 799–821. doi: 10.1038/s41596-019-0264-1 31942082

[B15] Colvin K. M. Irie Y. Tart C. S. Urbano R. Whitney J. C. Ryder C.. (2012). The Pel and Psl polysaccharides provide Pseudomonas aeruginosa structural redundancy within the biofilm matrix. Environ. Microbiol. 14, 1913–1928. doi: 10.1111/J.1462-2920.2011.02657.X 22176658 PMC3840794

[B16] Costa O. Y. A. Raaijmakers J. M. Kuramae E. (2018). Microbial extracellular polymeric substances: ecological function and impact on soil aggregation. Front. Microbiol. 9, 1636. 30083145 10.3389/fmicb.2018.01636 PMC6064872

[B17] Craig N. L. Craigie R. Gellert M. Lambowitz A. Editors. (2002). Mobile DNA II. American Society for Microbiology, Washington D.C.

[B18] Craig N. L. (2002). Transposases and integrases. Available at: www.els.net (Accessed April 13, 2023).

[B19] D’Angelo C. Casillo A. Melchiorre C. Lauro C. Corsaro M. M. Carpentieri A.. (2022). CATASAN is a new anti-biofilm agent produced by the marine antarctic bacterium psychrobacter sp. TAE2020. Mar. Drugs 20, 747. doi: 10.3390/MD20120747/S1 36547894 PMC9785100

[B20] Dass S. C. Wang R. (2022). Biofilm through the looking glass: A microbial food safety perspective. Pathogens (Basel, Switzerland) 11 (3), 346. doi: 10.3390/pathogens11030346 35335670 PMC8954374

[B21] De Gregorio E. Del Franco M. Martinucci M. Roscetto E. Zarrilli R. Di Nocera P. P. (2015). Biofilm-associated proteins: news from acinetobacter. BMC Genomics 16, 1–14. doi: 10.1186/S12864-015-2136-6/FIGURES/9 26572057 PMC4647330

[B22] Delmont T. O. Prestat E. Keegan K. P. Faubladier M. Robe P. Clark I. M.. (2012). Structure, fluctuation and magnitude of a natural grassland soil metagenome. ISME. J. 6, 1677. doi: 10.1038/ISMEJ.2011.197 22297556 PMC3498926

[B23] Ding W. Zhang W. Alikunhi N. M. Batang Z. Pei B. Wang R.. (2019). Metagenomic analysis of zinc surface–associated marine biofilms. Microb. Ecol. 77, 406–416. doi: 10.1007/s00248-018-01313-3 30612183

[B24] Donlan R. M. (2001). Biofilm formation: A clinically relevant microbiological process. Clin. Infect. Dis. 33, 1387–1392. doi: 10.1086/322972 11565080

[B25] Dwivedi V. Kumari K. Gupta S. K. Kumari R. Tripathi C. Lata P.. (2015). Thermus parvatiensis RLT sp. nov., Isolated from a Hot Water Spring, Located Atop the Himalayan Ranges at Manikaran, India. Indian J. Microbiol. 55, 357–365. doi: 10.1007/S12088-015-0538-4/TABLES/3 26543260 PMC4627951

[B26] Feldgarden M. Brover V. Gonzalez-Escalona N. Frye J. G. Haendiges J. Haft D. H.. (2021). AMRFinderPlus and the Reference Gene Catalog facilitate examination of the genomic links among antimicrobial resistance, stress response, and virulence. Sci. Rep. 11, 1–9. doi: 10.1038/s41598-021-91456-0 34135355 PMC8208984

[B27] Franklin M. J. Nivens D. E. Weadge J. T. Lynne Howell P. (2011). Biosynthesis of the pseudomonas aeruginosa extracellular polysaccharides, alginate, Pel, and Psl. Front. Microbiol. 2. doi: 10.3389/FMICB.2011.00167/BIBTEX PMC3159412 21991261

[B28] García-López M. L. Santos J. A. Otero A. Rodríguez-Calleja J. M. (2014). Psychrobacter. Encycl. Food Microbiol., 261–268. doi: 10.1016/B978-0-12-384730-0.00285-8

[B29] Giltner C. L. Nguyen Y. Burrows L. L. (2012). Type IV pilin proteins: versatile molecular modules. Microbiol. Mol. Biol. Rev. 76, 740. doi: 10.1128/MMBR.00035-12 23204365 PMC3510520

[B30] Guo J. Bolduc B. Zayed A. A. Varsani A. Dominguez-Huerta G. Delmont T. O.. (2021). VirSorter2: a multi-classifier, expert-guided approach to detect diverse DNA and RNA viruses. Microbiome 9, 1–13. doi: 10.1186/S40168-020-00990-Y/FIGURES/5 33522966 PMC7852108

[B31] Habimana O. Heir E. Langsrud S. Åsli A. W. Møretrø T. (2010). Enhanced surface colonization by escherichia coli 0157:H7 in biofilms formed by an acinetobacter calcoaceticus isolate from meat-processing environments. Appl. Environ. Microbiol. 76, 4557–4559. doi: 10.1128/AEM.02707-09/SUPPL_FILE/SUPPLEMENTARY_MATERIAL.DOC 20453142 PMC2897464

[B32] Hickman A. B. Dyda F. (2015). Mechanisms of DNA transposition. Microbiol. Spectr. 3, MDNA3. doi: 10.1128/MICROBIOLSPEC.MDNA3-0034-2014 PMC7422641 26104718

[B33] Hinsa S. M. Espinosa-Urgel M. Ramos J. L. O’Toole G. A. (2003). Transition from reversible to irreversible attachment during biofilm formation by Pseudomonas fluorescens WCS365 requires an ABC transporter and a large secreted protein. Mol. Microbiol. 49, 905–918. doi: 10.1046/J.1365-2958.2003.03615.X 12890017

[B34] Hinsa-Leasure S. M. Koid C. Tiedje J. M. Schultzhaus J. N. (2013). Biofilm formation by Psychrobacter arcticus and the role of a large adhesin in attachment to surfaces. Appl. Environ. Microbiol. 79, 3967–3973. doi: 10.1128/AEM.00867-13/ASSET/8A3C9854-687B-4BD4-B1F4-9228A1490A55/ASSETS/GRAPHIC/ZAM9991044790006.JPEG 23603675 PMC3697580

[B35] Iglewski B. H. (1996). Pseudomonas. Baron S. editor. Medical Microbiology. 4th edition, Chapter 27 Galveston (TX): University of Texas Medical Branch at Galveston. Available from: https://www.ncbi.nlm.nih.gov/books/NBK8326/.

[B36] Interagency Food Safety Analytics Collaboration (2022). Interagency Food Safety Analytics Collaboration. Foodborne illness source attribution estimates for 2020 for Salmonella, Escherichia coli O157, and Listeria monocytogenes using multi-year outbreak surveillance data, United States. Interag. Food Saf. Anal. Collab. 157.

[B37] Kanehisa M. Furumichi M. Tanabe M. Sato Y. Morishima K. (2017). KEGG: new perspectives on genomes, pathways, diseases and drugs. Nucleic Acids Res. 45, D353–D361. doi: 10.1093/NAR/GKW1092 27899662 PMC5210567

[B38] Keegan K. P. Glass E. M. Meyer F. (2016). MG-RAST, a metagenomics service for analysis of microbial community structure and function. Methods Mol. Biol. 1399, 207–233. doi: 10.1007/978-1-4939-3369-3_13/FIGURES/9 26791506

[B39] Kim M. Rodriguez-R L. M. Hatt J. K. Kayali O. Nalá R. Dunlop A. L.. (2022). Higher pathogen load in children from Mozambique vs. USA revealed by comparative fecal microbiome profiling. ISME. Commun. 2, 74. doi: 10.1038/s43705-022-00154-z PMC9723681 37938667

[B40] Kline K. A. Fälker S. Dahlberg S. Normark S. Henriques-Normark B. (2009). Bacterial adhesins in host-microbe interactions. Cell Host Microbe 5, 580–592. doi: 10.1016/J.CHOM.2009.05.011 19527885

[B41] Lavire C. Normand P. Alekhina I. Bulat S. Prieur D. Birrien J. L.. (2006). Presence of Hydrogenophilus thermoluteolus DNA in accretion ice in the subglacial Lake Vostok, Antarctica, assessed using rrs, cbb and hox. Environ. Microbiol. 8, 2106–2114. doi: 10.1111/J.1462-2920.2006.01087.X 17107552

[B42] Li H. Durbin R. (2009). Fast and accurate short read alignment with Burrows–Wheeler transform. Bioinformatics 25, 1754–1760. doi: 10.1093/BIOINFORMATICS/BTP324 19451168 PMC2705234

[B43] Li W. Godzik A. (2006). Cd-hit: A fast program for clustering and comparing large sets of protein or nucleotide sequences. Bioinformatics (Oxford, England) 22 (13), 1658–1659. doi: 10.1093/bioinformatics/btl158 16731699

[B44] Li D. Liu C. M. Luo R. Sadakane K. Lam T. W. (2015). MEGAHIT: An ultra-fast single-node solution for large and complex metagenomics assembly *via* succinct de Bruijn graph. Bioinformatics 31, 1674–1676. doi: 10.1093/bioinformatics/btv033 25609793

[B45] Limoli D. H. Jones C. J. Wozniak D. J. (2015). Bacterial extracellular polysaccharides in biofilm formation and function. Microbiol. Spectr. 3 (3). doi: 10.1128/MICROBIOLSPEC.MB-0011-2014 PMC4657554 26185074

[B46] Louca S. Jacques S. M. S. Pires A. P. F. Leal J. S. Srivastava D. S. Parfrey L. W.. (2016). High taxonomic variability despite stable functional structure across microbial communities. Nat. Ecol. Evol. 1 (1), 1–12. doi: 10.1038/s41559-016-0015 28812567

[B47] Louca S. Polz M. F. Mazel F. Albright M. B. N. Huber J. A. O’Connor M. I.. (2018). Function and functional redundancy in microbial systems. Nat. Ecol. Evol. 2 (6), 936–943. doi: 10.1038/s41559-018-0519-1 29662222

[B48] Luo Y. H. Lai Y. J. S. Zheng C. Ilhan Z. E. Ontiveros-Valencia A. Long X.. (2021). Increased expression of antibiotic-resistance genes in biofilm communities upon exposure to cetyltrimethylammonium bromide (CTAB) and other stress conditions. Sci. Total. Environ. 765, 144264. doi: 10.1016/J.SCITOTENV.2020.144264 33418325

[B49] Madigan M. T. Martinko J. M. David A. Stahl D. P. C. (2011). Brock biology of microorganisms (Pearson Education). doi: 10.1093/nq/s3-XII.310.469-a

[B50] Madsen J. S. Burmølle M. Hansen L. H. Sørensen S. J. (2012). The interconnection between biofilm formation and horizontal gene transfer. FEMS Immunol. Med. Microbiol. 65, 183–195. doi: 10.1111/J.1574-695X.2012.00960.X 22444301

[B51] McMurdie P. J. Holmes S. (2013). Phyloseq: an R package for reproducible interactive analysis and graphics of microbiome census data. PloS One 8 (4), e61217. doi: 10.1371/journal.pone.0061217 23630581 PMC3632530

[B52] Minamino T. Morimoto Y. V. Hara N. Namba K. (2011). An energy transduction mechanism used in bacterial flagellar type III protein export. Nat. Commun. 2, 1–9. doi: 10.1038/ncomms1488 PMC3195256 21934659

[B53] Mitchell H. L. Dashper S. G. Catmull D. V. Paolini R. A. Cleal S. M. Slakeski N.. (2010). Treponema denticola biofilm-induced expression of a bacteriophage, toxin-antitoxin systems and transposases. Microbiology 156, 774–788. doi: 10.1099/MIC.0.033654-0/CITE/REFWORKS 20007650

[B54] Muriel C. Jalvo B. Redondo-Nieto M. Rivilla R. Martín M. (2015). Chemotactic Motility of Pseudomonas fluorescens F113 under Aerobic and Denitrification Conditions. PloS One 10 (7), 0132242. doi: 10.1371/JOURNAL.PONE.0132242 PMC4498747 26161531

[B55] Nobile C. J. Andes D. R. Nett J. E. Smith F. J. Yue F. Phan Q.-T.. (2006). Critical role of bcr1-dependent adhesins in C. albicans biofilm formation *in vitro* and *in vivo* . PloS Pathog. 2 (7), e63. doi: 10.1371/JOURNAL.PPAT.0020063 16839200 PMC1487173

[B56] O’Toole G. A. Kolter R. (1998). Initiation of biofilm formation in Pseudomonas fluorescens WCS365 proceeds *via* multiple, convergent signalling pathways: a genetic analysis. Mol. Microbiol. 28, 449–461. doi: 10.1046/J.1365-2958.1998.00797.X 9632250

[B57] Overbeek R. Olson R. Pusch G. D. Olsen G. J. Davis J. J. Disz T.. (2014). The SEED and the Rapid Annotation of microbial genomes using Subsystems Technology (RAST). Nucleic Acids Res. 42, D206. doi: 10.1093/NAR/GKT1226 24293654 PMC3965101

[B58] Packiavathy I. A. S. V. Kannappan A. Thiyagarajan S. Srinivasan R. Jeyapragash D. Paul J. B. J.. (2021). AHL-lactonase producing psychrobacter sp. From palk bay sediment mitigates quorum sensing-mediated virulence production in gram negative bacterial pathogens. Front. Microbiol. 12. doi: 10.3389/FMICB.2021.634593/BIBTEX PMC8079732 33935995

[B59] Parks D. H. Imelfort M. Skennerton C. T. Hugenholtz P. Tyson G. W. (2015). CheckM: assessing the quality of microbial genomes recovered from isolates, single cells, and metagenomes. Genome Res. 25, 1043. doi: 10.1101/GR.186072.114 25977477 PMC4484387

[B60] Pratt L. A. Kolter R. (1999). Genetic analyses of bacterial biofilm formation. Curr. Opin. Microbiol. 2, 598–603. doi: 10.1016/S1369-5274(99)00028-4 10607630

[B61] Rainey K. Michalek S. M. Wen Z. T. Wu H. (2018). Glycosyltransferase-mediated biofilm matrix dynamics and virulence of Streptococcus mutans. Appl. Environ. Microbiol. 85 (5), e02247-18. doi: 10.1128/AEM.02247-18/SUPPL_FILE/AEM.02247-18-S0001.PDF PMC6384114 30578260

[B62] Rees D. C. Johnson E. Lewinson O. (2009). ABC transporters: the power to change. Nat. Rev. Mol. Cell Biol. 10, 218–227. doi: 10.1038/nrm2646 19234479 PMC2830722

[B63] Rehman Z. U. Fortunato L. Cheng T. Leiknes T. O. (2020). Metagenomic analysis of sludge and early-stage biofilm communities of a submerged membrane bioreactor. Sci. Total. Environ. 701, 134682. doi: 10.1016/j.scitotenv.2019.134682 31704413

[B64] Robertson J. Nash J. H. E. (2018). MOB-suite: software tools for clustering, reconstruction and typing of plasmids from draft assemblies. Microb. Genomics 4 (8), e000206. doi: 10.1099/MGEN.0.000206 PMC6159552 30052170

[B65] Skipper P. J. A. Skipper L. K. Dixon R. A. (2022). A metagenomic analysis of the bacterial microbiome of limestone, and the role of associated biofilms in the biodeterioration of heritage stone surfaces. Sci. Rep. 12, 1–18. doi: 10.1038/s41598-022-08851-4 35318388 PMC8940931

[B66] Song W. Wemheuer B. Steinberg P. D. Marzinelli E. M. Thomas T. (2021). Contribution of horizontal gene transfer to the functionality of microbial biofilm on a macroalgae. ISME. J. 15, 807–817. doi: 10.1038/s41396-020-00815-8 33558686 PMC8027169

[B67] Srey S. Jahid I. K. Ha S. Do (2013). Biofilm formation in food industries: A food safety concern. Food Control. 31, 572–585. doi: 10.1016/J.FOODCONT.2012.12.001

[B68] Stellato G. Utter D. R. Voorhis A. De Angelis M. Murat Eren A. Ercolini D. (2017). A few Pseudomonas oligotypes dominate in the meat and dairy processing environment. Front. Microbiol. 8. doi: 10.3389/FMICB.2017.00264/FULL PMC5332365 28303120

[B69] Sun G. (2011). Antibacterial textile materials for medical applications. Funct. Text. Improv. Performance. Prot. Heal., 360–375. doi: 10.1533/9780857092878.360

[B70] Tao F. Swarup S. Zhang L. H. (2010). Quorum sensing modulation of a putative glycosyltransferase gene cluster essential for Xanthomonas campestris biofilm formation. Environ. Microbiol. 12, 3159–3170. doi: 10.1111/J.1462-2920.2010.02288.X 20636376

[B71] Theilacker C. Sava I. Sanchez-Carballo P. Bao Y. Kropec A. Grohmann E.. (2011). Deletion of the glycosyltransferase bgsB of Enterococcus faecalis leads to a complete loss of glycolipids from the cell membrane and to impaired biofilm formation. BMC Microbiol. 11, 1–11. doi: 10.1186/1471-2180-11-67/TABLES/2 21470413 PMC3083329

[B72] Tian L. Wang X. W. Wu A. K. Fan Y. Friedman J. Dahlin A.. (2020). Deciphering functional redundancy in the human microbiome. Nat. Commun. 11 (1), 1–11. doi: 10.1038/s41467-020-19940-1 33277504 PMC7719190

[B73] Tu Q. He Z. Wu L. Xue K. Xie G. Chain P.. (2017). Metagenomic reconstruction of nitrogen cycling pathways in a CO2-enriched grassland ecosystem. Soil Biol. Biochem. 106, 99–108. doi: 10.1016/J.SOILBIO.2016.12.017

[B74] Turnbaugh P. J. Hamady M. Yatsunenko T. Cantarel B. L. Duncan A. Ley R. E.. (2008). A core gut microbiome in obese and lean twins. Nature 457 (7228), 480–484. doi: 10.1038/nature07540 19043404 PMC2677729

[B75] Tyson G. W. Chapman J. Hugenholtz P. Allen E. E. Ram R. J. Richardson P. M.. (2004). Community structure and metabolism through reconstruction of microbial genomes from the environment. Nat 428, 37–43. doi: 10.1038/nature02340 14961025

[B76] Van Houdt R. Michiels C. W. (2010). Biofilm formation and the food industry, a focus on the bacterial outer surface. J. Appl. Microbiol. 109, 1117–1131. doi: 10.1111/J.1365-2672.2010.04756.X 20522145

[B77] Voglauer E. M. Zwirzitz B. Thalguter S. Selberherr E. Wagner M. Rychli K. (2022). Biofilms in water hoses of a meat processing environment harbor complex microbial communities. Front. Microbiol. 13. doi: 10.3389/fmicb.2022.832213 PMC8882869 35237250

[B78] Wang R. Dass S. C. Chen Q. Guragain M. Bosilevac J. M. Wang R.. (2022). Characterization of salmonella strains and environmental microorganisms isolated from a meat plant with salmonella recurrence. Meat. Muscle Biol. 6, 1–11. doi: 10.22175/MMB.15442

[B79] Wang G. Li J. Xie S. Zhai Z. Hao Y. (2020). The N-terminal domain of rhamnosyltransferase EpsF influences exopolysaccharide chain length determination in Streptococcus thermophilus 05-34. PeerJ 8, e8524. doi: 10.7717/peerj.8524 PMC7023835 32095353

[B80] Welter D. K. Ruaud A. Henseler Z. M. De Jong H. N. Van Coeverden De Groot P. Michaux J.. (2021). Free-living, psychrotrophic bacteria of the genus psychrobacter are descendants of pathobionts. mSystems 6 (2), e00258-21. doi: 10.1128/mSystems.00258-21 33850039 PMC8546975

[B81] Wickham H. (2016). ggplot2 elegant graphics for data analysis (Springer International Publishing). doi: 10.1007/978-3-319-24277-4

[B82] Wood D. E. Lu J. Langmead B. (2019). Improved metagenomic analysis with Kraken 2. Genome Biol. 20, 1–13. doi: 10.1186/S13059-019-1891-0/FIGURES/2 31779668 PMC6883579

[B83] Wu Y. W. Simmons B. A. Singer S. W. (2016). MaxBin 2.0: an automated binning algorithm to recover genomes from multiple metagenomic datasets. Bioinformatics 32, 605–607. doi: 10.1093/BIOINFORMATICS/BTV638 26515820

[B84] Yadav B. S. Ronda V. Vashista D. P. Sharma B. (2013). Sequencing and computational approaches to identification and characterization of microbial organisms. Biomed. Eng. Comput. Biol. 5, 43. doi: 10.4137/BECB.S10886 25288901 PMC4147756

[B85] Yang X. Noyes N. R. Doster E. Martin J. N. Linke L. M. Magnuson R. J.. (2016). Use of metagenomic shotgun sequencing technology to detect foodborne pathogens within the microbiome of the beef production chain. Appl. Environ. Microbiol. 82, 2433–2443. doi: 10.1128/AEM.00078-16 26873315 PMC4959480

[B86] Yin W. Wang Y. Liu L. He J. (2019). Biofilms: the microbial “Protective clothing” in extreme environments. Int. J. Mol. Sci. 20 (14), 3423. doi: 10.3390/IJMS20143423 31336824 PMC6679078

[B87] Zhou X. Bi X. Yang T. Fan X. Shi X. Wang L.. (2022). Metagenomic insights into microbial nitrogen metabolism in two-stage anoxic/oxic-moving bed biofilm reactor system with multiple chambers for municipal wastewater treatment. Bioresour. Technol. 361, 127729. doi: 10.1016/J.BIORTECH.2022.127729 35931282

[B88] Zhu Z. Wu C. Zhong D. Yuan Y. Shan L. Zhang J. (2014). Effects of pipe materials on chlorine-resistant biofilm formation under long-term high chlorine level. Appl. Biochem. Biotechnol. 173, 1564–1578. doi: 10.1007/S12010-014-0935-X/FIGURES/7 24828580

[B89] Zhu L. Yuan H. Shi Z. Deng L. Yu Z. Li Y.. (2022). Metagenomic insights into the effects of various biocarriers on moving bed biofilm reactors for municipal wastewater treatment. Sci. Total. Environ. 813, 151904. doi: 10.1016/J.SCITOTENV.2021.151904 34838558

