# Supplementary figures and images for: Shotgun-metagenomics reveals a highly diverse and communal microbial network present in the drains of three beef-processing plants
Source: Front Cell Infect Microbiol. 2023 Sep 8;13:1240138. doi: 10.3389/fcimb.2023.1240138 (PMC10515220; doi:10.3389/fcimb.2023.1240138)

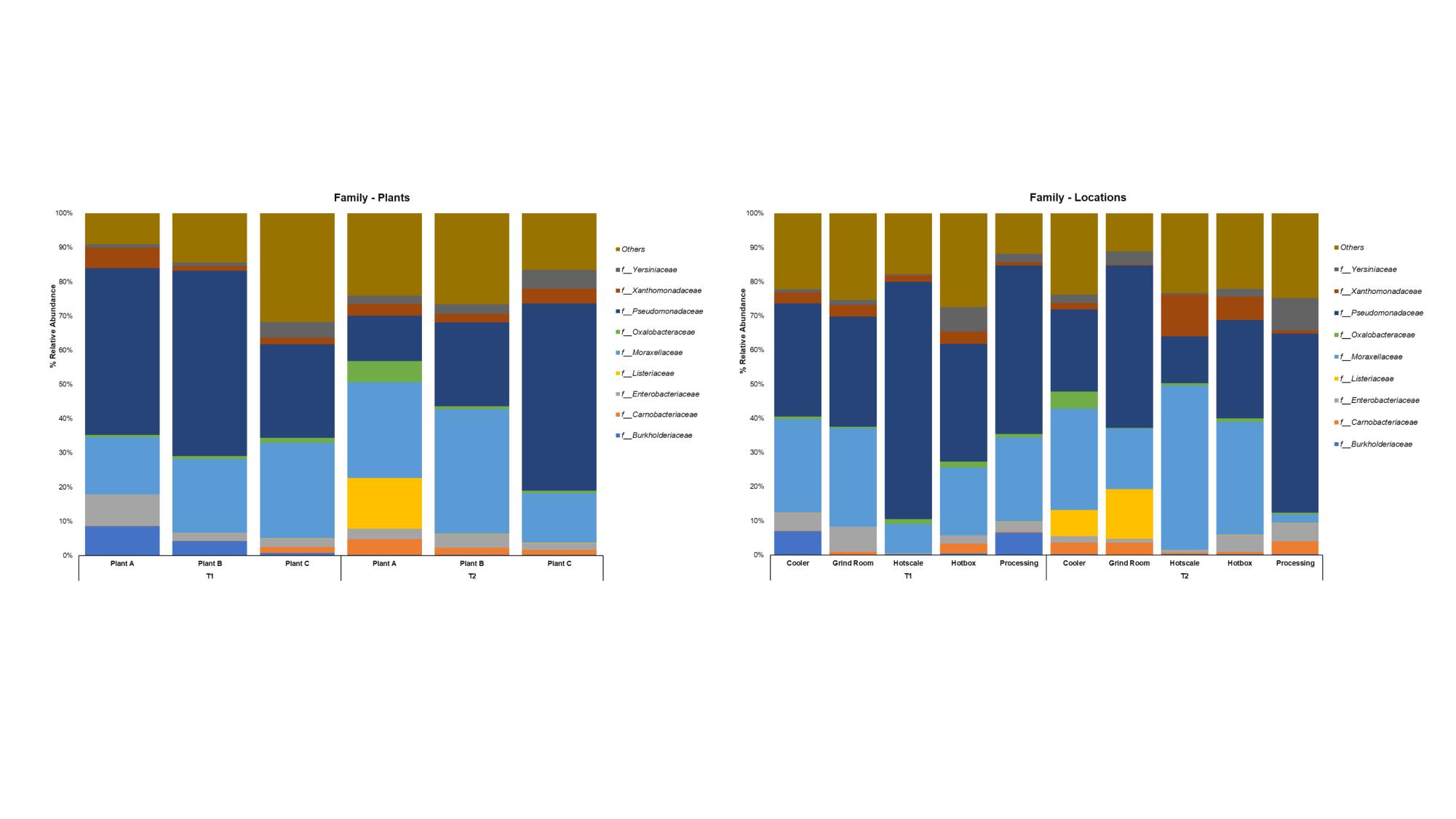

Supplement: Supplementary Figure S1 [file Image_1.jpg]

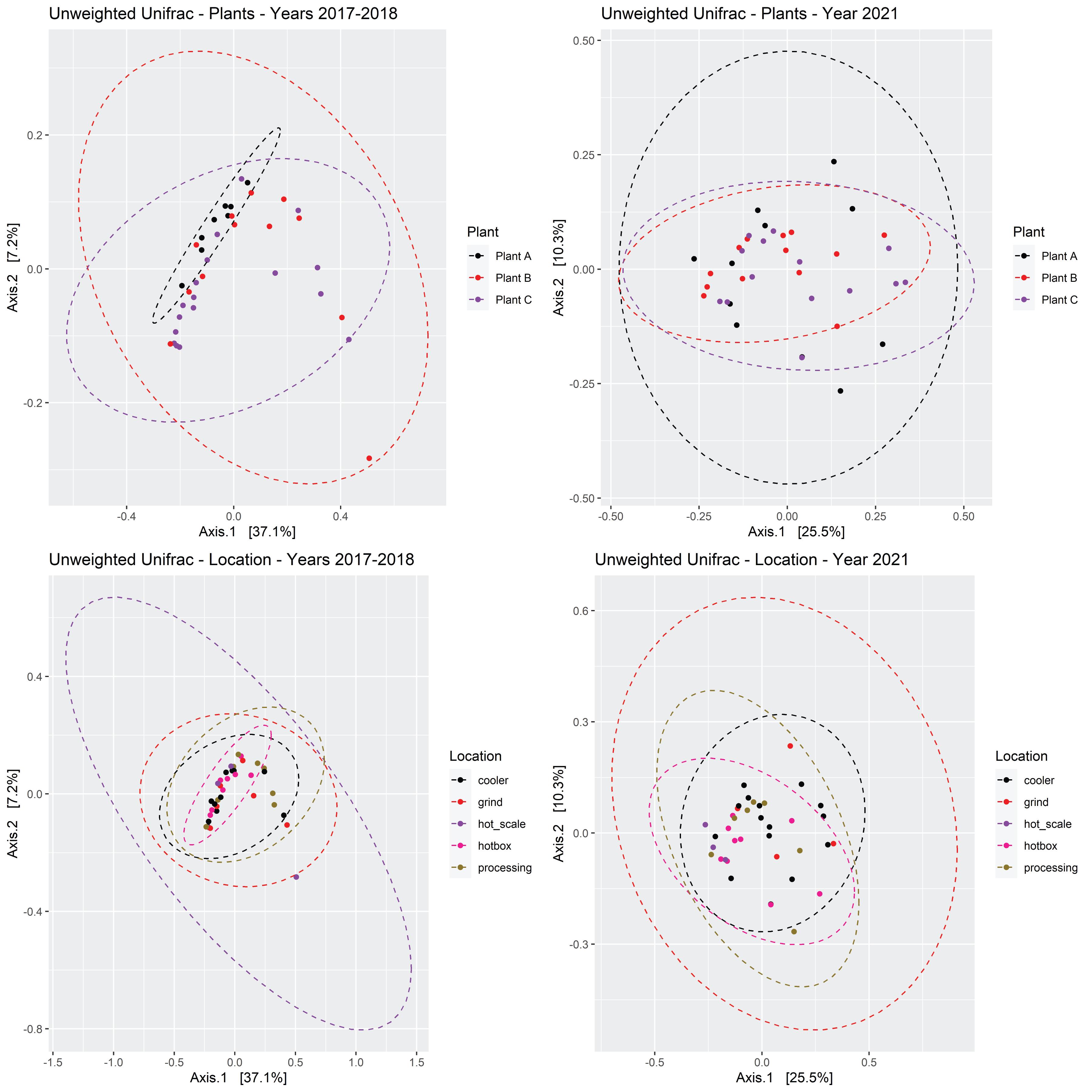

Supplement: Supplementary Figure S2 [file Image_2.png]

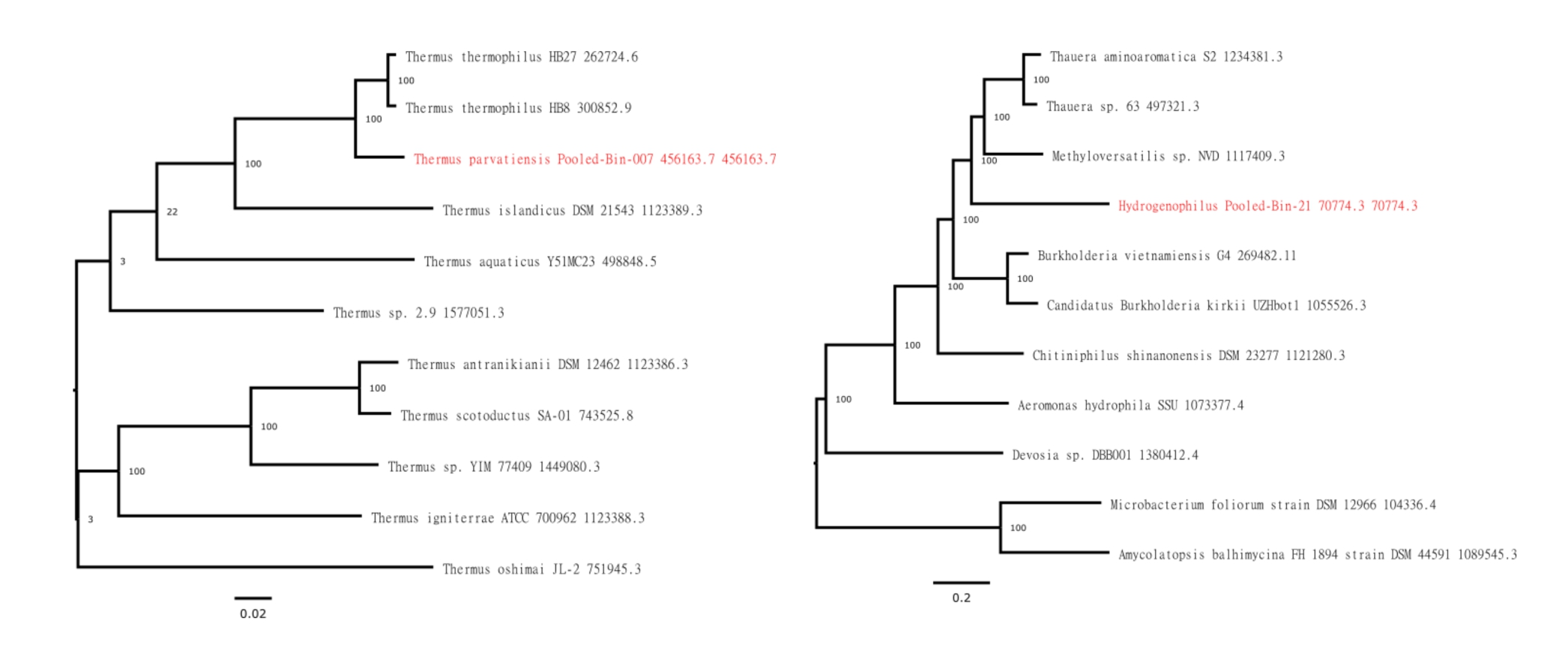

Supplement: Supplementary Figure S3 [file Image_3.jpg]
